# (Re)constructing Intersectional Masculinities Following Myocardial Infarction

**DOI:** 10.1111/1467-9566.70151

**Published:** 2026-02-09

**Authors:** Jesper Andreasson, Thomas Johansson, Carina Danemalm‐Jägervall, Anna Strömberg

**Affiliations:** ^1^ Department of Sport Science Linnaeus University Kalmar Sweden; ^2^ Department of Education, Communication and Learning University of Gothenburg Gothenburg Sweden; ^3^ Region Kronoberg Växjö Sweden; ^4^ Department of Health Medicine, and Caring Sciences Linköping Sweden; ^5^ Department of Cardiology University of Linköping Linköping Sweden

## Abstract

This study builds on interview data with 24 Swedish men who have been diagnosed with and treated for myocardial infarction (MI). The aim is to explore how masculine subjectivities are constituted and dynamically negotiated through men's meaning‐making of their illness experiences, with particular attention to how notions of masculinity intersect with and are shaped by social categories such as class and age. Across the data, various configurations of masculinity were generated: from stoic endurance and bodily control to emotional reflexivity and relational responsibility. These constructs of masculinity are not equally valued, however. Certain seemingly masculine ideals, such as autonomy and productivity, tend to remain culturally dominant, whereas other modes, such as care orientation, are more conditionally addressed. Furthermore, the results show how masculinity, in intersection with age and socioeconomic position, may function as both an impediment and an opportunity for dealing with the disruption that the MI might bring in terms of changes in lifestyle, health, work and family life. By doing so, the study contributes to theorising hegemonic masculinity, showing how it is continuously reconfigured rather than fixed and how it is negotiated through classed and aged subject positions in the context of illness.

## 1 Introduction

Cultural ideals of masculinity have long been associated with strength, independence, authority and control—traits often emphasised at the expense of emotional expression and health awareness. Historically, men have been socialised to downplay illness and prioritise performance and stoicism over self‐care (Haywood et al. 2017). Stereotypical gender norms and scripts have been linked to risky health behaviours, including reluctance to seek care or express concerns about body image, mental health or sexuality (Gough and Novikova 2020; Mokhwelepa and Sumbane 2025; Piatkowski et al. 2023). However, notions of masculinity are neither fixed nor uniform. They are historically contingent and subject to redefinition across time and context.

Scholars suggest that contemporary forms of masculinity increasingly include self‐care, health awareness and emotional openness (Connell and Messerschmidt 2005; Elliot 2016; P. M. Galdas et al. 2023; Nordin et al. 2024). In this way, masculinity can be seen as positioned at the intersection of continuity and change. This discussion has often been situated in relation to hegemonic masculinity and further pursued through the exploration and conceptualisation of other forms of masculinities (see, e.g., Anderson 2009; Elliot 2016; Gater 2023). In this study, when discussing masculinity, health and illness, we are, however, not primarily concerned with different forms of masculinities per se, but rather with the ongoing reconfiguration of hegemonic masculinity itself and how different forms of masculinity are intertwined with other social categories such as class and age. Given this analytical orientation, masculinity constitutes the primary conceptual focus of the article, whereas we use myocardial infarction (MI), commonly known as a heart attack and defined as the interruption of blood flow to the heart muscle resulting in tissue damage, as the empirical example/context through which masculine subjectivities are articulated, challenged and renegotiated.

Although gendered patterns of masculinity are culturally and socially constructed, they have tangible and significant consequences for men's health behaviour and experience. In the context of MI, in which early symptom recognition and emotional support are crucial, prevailing gender norms shape how men interpret bodily signals and engage (or not) with healthcare systems (cf. Hwang et al. 2022). Broadly, MI is one of the most common causes of death globally (Nowbar et al. 2019; Salari et al. 2023). At the same time, there is significant variation within patient groups in terms of who tends to be affected and when they are affected. MI in particular—and cardiovascular disease in general—is notably more prevalent among men, older adults and individuals with lower educational attainment (Khaing et al. 2017; Salari et al. 2023). Risk factors are thus gender, age and socioeconomic status, as well as comorbid conditions, such as diabetes, hyperlipidaemia and hypertension. In addition, MI is fundamentally associated with lifestyle factors, among which physical inactivity, unhealthy diet, psychological stress, insufficient social support and tobacco use are primary (Vrints et al. 2024).

Despite extensive knowledge of epidemiology and risk factors, there remains a significant gap in our understanding of how experiencing MI influences men's self‐perceptions and subject positions—particularly from intersectional perspectives. Integrating an intersectional perspective on masculinities and cardiovascular disease is essential for understanding how gender norms intersect with other social factors to shape health behaviour, risk and access to care. Following this, the current article adopts a qualitative research approach based on interviews with Swedish men who have experienced MI. *The aim is to explore how masculine subjectivities are constituted and dynamically negotiated through men's meaning‐making of their illness experiences, with particular attention to how masculinity intersects with and is shaped by social categories such as class and age.* Drawing on an intersectional perspective, the analysis examines these categories as both lived experiences and discursive resources in the ongoing production of gendered identities across different stages of the illness trajectory. To capture two interconnected stages of this trajectory (the acute onset of MI and the longer‐term reflections on its consequences), we ask the following research questions (RQs):

RQ1 How do men treated for MI narrate the onset of their illness and their decisions surrounding seeking medical care?

RQ2 How do men reflect on the implications of MI for their health, lifestyle and family relationships, and how do these reflections articulate negotiations of masculinity in relation to intersecting social categories such as class and age?

## 2 Background and Literature

Although there is a growing literature on masculinity and cardiovascular disease in the social sciences, to date research on patient experience is found mainly in the social medicine and caring sciences literature. Critical scholars, however, have noted that the dominance of biomedical perspectives, unsurprisingly, tends to foreground pathological framings of MI, thereby narrowing the discursive space for more holistic perspectives. Such framings risk marginalising the emotional, social and existential dimensions of the illness while also obscuring how gender—intersecting with factors such as age and class—shapes individuals' experience of illness and recovery (Wemrell et al. 2017).

Regarding gender, indeed the role of masculinity as a predictor of MI severity was investigated some decades ago (Helgeson 1990). There is a vast literature from the later part of the previous century paying attention to the link between Type A behaviour (aggressiveness, achievement orientation and easily aroused hostility) and MI. In a similar vein, a longitudinal British study showed that higher ‘femininity’ scores in men were associated with lower risk of death after coronary heart disease (Hunt et al. 2007; Lohse et al. 2017). However, there is also a long tradition of criticism of these kinds of studies and their attempts to predict MI using personality traits, which have been deemed outdated (see Riska 2002). Instead, scholars have more recently paid attention to how men experience a cardiac event and how they cope with the emotional challenges that result from their disease (Emslie and Hunt 2009; Jbilou et al. 2019; Smith et al. 2022). Jbilou et al. (2019), for example, followed 93 men who were diagnosed with MI, unstable angina or who had had heart surgery and showed that many of the participants had purposely delayed their hospital visit and ignored early symptoms (cf. Gough and Novikova 2020). Returning home after the cardiac event, the men also had difficulty complying with the advice to ‘slow down’ and ‘stay at home’ (read: breaking with normative masculinity configurations) (see also Mokhwelepa and Sumbane 2025). Moreover, sexual difficulties after the cardiac event were frequently reported (see also Liu et al. 2016; Smith et al. 2022).

Although a substantial body of literature underscores the importance of gender‐sensitive approaches to cardiac disease—highlighting conventional forms of masculinity as a potential barrier to help‐seeking, resilience, coping and lifestyle change—it is of relevance to note that there is also growing recognition of the need to explore points of convergence in how men and women experience and respond to the illness. This includes calls for more consistent use of intersectional perspectives that account for how gender interacts with categories such as age, class and education (see, e.g., Pelletier et al. 2015; Wemrell et al. 2017; Willis et al. 2023). P. M. Galdas et al. (2010) argue that men's and women's decisions to seek or delay seeking treatment for acute cardiac symptoms cannot be easily categorised as masculine or feminine strategies, but that there may be other factors that influence such decisions across (and within) genders (see also Elliot 2016). In a study of male survivors of sudden cardiac arrest and their partners, Uren and Galdas (2015) also found that although some of the men aligned with and embodied self‐reliance, strength and denial of weakness, others could reframe their masculinity and become more responsible and self‐reflective in relation to their disease (see also Nordin et al. 2024).

Scholars have also approached MI using intersectional perspectives. In a Canadian study of men going through exercise‐based cardiac rehabilitation programmes, Dale et al. (2015) found that masculinity intersects with age, employment status and class. Older and retired men, for example, showed a greater ability to attend rehabilitation and adopt health recommendations in comparison with younger breadwinner men/masculinities (see also P. Galdas et al. 2007; P. M. Galdas and Cheater 2010). In relation to this, Taylor Smith and Dumas (2019) suggest that men in economically advanced societies are not only more at risk of dying prematurely due to heart disease than women but also that this risk is related to socioeconomic status and masculinity.

Following this, paying attention to the social stratification of the risk of heart disease, Wemrell et al. (2017) have suggested that intersectionality theory can contribute to public health by increasing knowledge about heterogeneities between and within social categories, widening our understanding of how health disparities are produced through different power dynamics, including age, gender and class. This study suggests that risk differences between social groups are better met by changes in care related to macrolevel or mesolevel factors rather than by interventions aiming to change individual behaviour (thus somewhat breaking with the idea of person‐centred care, i.e., to be understood as paradigmatic in Swedish healthcare) (cf. P. M. Galdas et al. 2023). The study further emphasises the significance of intersectional approaches and analyses to address such changes—for example, through policies developed to improve public health.

In summary, hegemonic notions of masculinity have been identified as a risk factor for MI, with growing scholarship examining gender and heart disease. Less attention has, however, been given to how intersecting social positions shape help‐seeking behaviours across the care process. This highlights the importance of culturally sensitive and theoretically grounded approaches that investigate how hegemonic and plural masculinities are shaped by age and socioeconomic context. Socioeconomic status remains a key determinant of disparities in men's health behaviours and outcomes, contributing to higher MI risk and premature mortality compared to women (Taylor Smith and Dumas 2019). Although men show a higher overall MI prevalence, significant variation exists across socioeconomic and age groups. Thus, we argue, greater attention to intersectional perspectives and masculinities is essential to understanding MI risk and recovery—an area to which this article seeks to contribute.

## 3 Masculinity Theory and Intersectionality

Theoretically, this study is situated within the field of critical studies on men and masculinities. It engages with ongoing debates on the configurations of hegemonic masculinity, emphasising the plurality and relational nature of masculinities (Connell 1995; Haywood et al. 2017). According to Connell, men's emotional lives, health and bodies are structured hierarchically, wherein certain masculinities and embodiments are more highly valued and culturally normative than others. Consequently, men's subject positions are differentially organised and dominated by what Connell terms hegemonic masculinity. Conceptually, hegemonic masculinity refers both to the culturally dominant ideal of masculinity within a given context and to the mechanisms by which the existing gender order is sustained—and at times contested. Although there is considerable variation in how masculinity is understood and enacted, it is commonly approached as something performed or done relationally, through practices that are situated in relation to other men and bodies (Hearn 2004; Kimmel 2008; Reeser and Gottzén 2018; Robertson 2007).

Taking a sociological approach, this article explores how understandings of masculinity, health and lifestyle are articulated by male patients with MI in meaning‐making processes that contribute to the production of common‐sense notions of masculinity, masculine bodies and coronary disease. The analysis foregrounds a nuanced, phenomenological and embodied perspective on the relationship between heart conditions, masculinity and surrounding social and cultural expectations. On a sociocultural level, men's narratives are situated within dominant discourses of hegemonic masculinity, which shape how they respond to physical changes resulting from MI and, potentially, from ageing. On an inter‐relational level, masculine subjectivities are negotiated through interactions with healthcare professionals, as well as within intimate relationships.

In this article, we apply an intersectional perspective with a central focus on masculinity while also considering how age and class intersect to shape experiences of the ill body. Intersectionality theories provide a framework to understand how multiple social categories simultaneously influence subjectivity, belonging and power relations. Here, masculinity is understood as a fluid and unstable social category that is continuously reshaped through its intersections with other categories (Christensen and Qvotrup‐Jensen 2014). Age, for example, can be understood, rather than simply as chronological time, as a social category tied to life phases such as adulthood and older age, each carrying distinct social and cultural expectations and meanings that influence masculine subjectivities (Sandberg 2016). Similarly, class is here addressed as ‘something’ that shapes access to resources, social positioning and lived experience, which (again), in turn, affect how gendered subject positions are performed and negotiated. In the analysis, we focus on the everyday meaning‐making processes in which masculinity, age and class are articulated and mobilised as discursive resources. We argue that our participants actively use these categories not only to position themselves and others within culturally available masculine subject positions but also in shaping how masculinity is experienced and lived in relation to (their) illness and recovery.

## 4 Method and Research Design

This study has a qualitative design and builds on a sample of 24 Swedish men aged 38–79 (mean age 57) who were diagnosed with and treated for MI between 2023 and 2025. The sample reflects diversity not only in terms of age but also employment status, with some participants being actively employed and others retired. Ten of the men had a working‐class background and 14 a middle‐class background. Class position was assessed through participants' educational attainment, occupational history and how they described their economic circumstances. Drawing on an intersectional perspective, we conceptualise class as a socially situated and relational category, shaped through its intersection with other axes of power. Accordingly, class is not treated as a fixed attribute but as a dynamic and context‐dependent social position. All of the participants self‐identified as White. The participants had generally undergone percutaneous coronary intervention (PCI) and, in some cases, stent implantation to restore coronary artery flow, after which the majority had been discharged in stable condition. However, in some cases, the MI had been complicated by cardiac arrest, stent thrombosis and heart failure, leading to a more protracted and complex clinical course.

The data collection was facilitated by the clinical positions of two of the authors, one (CDJ) being employed as a sexologist at a county hospital and the other (AS) as a cardiac nurse at a university hospital. Their professional roles provided direct access to the target population—men who had experienced MI—and enabled a purposive sampling strategy aimed at including participants with diverse backgrounds, as described. The recruitment process was guided by an aim to reflect variations across relevant intersectional dimensions. However, despite targeted efforts, the recruitment of participants with minoritised ethnic backgrounds proved challenging, which limited the diversity of the sample in this regard (e.g., P. Galdas et al. 2007).

Interviews were conducted in designated private rooms in each hospital setting. These locations were specifically chosen to ensure confidentiality and provide an environment in which participants could speak freely without the risk of being overheard. A semi‐structured biographical interview approach was employed, drawing on the framework developed by Hallqvist and Hydén (2012). This approach reflected our aim of engaging in dialogue with the participants by exploring their illness narratives through storytelling and the chronological ordering of significant life events. The interviews focused on several key themes, including participants' accounts of the acute phase of their illness, their experience of diagnosis and treatment, and the impact of their illness on their self‐perception, daily life and social relationships.

Although a thematic interview guide was used, we prioritised attending to the participants' own narratives and the meanings they ascribed to their experiences. The structure of the guide was therefore applied loosely, and participants were not interrupted or redirected if their stories deviated from the order of the questions. To this end, we viewed ‘empathic listening’ (Back 2007) as essential to creating a setting in which participants felt sufficiently safe to share their experiences openly. Our intention was to listen as unconditionally as possible and to provide a supportive, nonthreatening environment throughout the interview process. This also involved acknowledging and critically reflecting on our own positionalities and how these may have shaped both the interview situation and the analysis (Aull Davies 2008). The interviews, stretching between 45 and 90 min in length, were recorded and transcribed verbatim.

When analysing the data, we followed the ‘listening guide’ as described by Walby (2012), which offers a relational and processual approach to narrative analysis. Central to this method is the idea that subjectivity is formed in and through relationships and that voices are multiple, shifting and embedded in social contexts. In our study, this framework enabled us to trace how men narrated the experience of MI in ways that reflected—and at times contradicted—normative understandings of masculinity, while also being shaped by class background and age. The analytical process unfolded through four interrelated steps:

*First*, we identified the core narrative or ‘plotline’ in each interview. This step involved listening closely to how participants made sense of being diagnosed with MI—how they narrated the disruption, reorientation and emotional impact of the event. *Second*, we focused on how participants constructed their sense of self in relation to the illness. This involved tracing the voice of the ‘I’ in each narrative and examining how it shifted across different parts of the story. *Third*, we listened for multiple voices within the same narrative—voices that reflected internal dialogues, conflicts or shifts in identity positions. *Finally*, we connected these personal narratives to broader cultural and structural discourses. This involved analysing how the participants' stories were shaped by, and responded to, dominant ideals of masculinity and health. Furthermore, we interpreted their reflections on masculinity in relation to their understandings of norms and values connected to age and class.

The intertwining of analysis and writing reflects both the processual character of the ‘listening guide’ (Walby 2012) and our broader qualitative and theoretically informed approach. This analytic orientation understands meaning‐making as unfolding in the dynamic interplay between listening, interpreting and writing. All potentially identifying details occurring in the text have been pseudonymised. Formal ethical approval to carry out the study was given by the Swedish Ethical Review Authority (Ref. No. 2023‐07225‐01).

## 5 Findings

This section presents the study's findings. The first analytic theme addresses the onset of the illness and participants' initial engagements with healthcare. The second theme explores the broader ramifications of the illness for the men's health, lifestyle and family life, examined along intersectional dimensions. Together, these themes directly engage with the research questions outlined earlier.

### 5.1 Seeking Care—Delayed Action Due To Bodily Unawareness and Stoic Masculinity

Although there is variation in the data, a salient and consistent theme in the narratives is a notable delay in the participants' help‐seeking behaviour. This is not surprising. Patterns of delayed help‐seeking and unawareness of bodily signs of cardiac illness are well documented in contemporary research (Ericsson et al. 2022; Gough and Novikova 2020). Numerous studies have shown how men tend to disregard bodily symptoms as a reflection of dominant masculine norms that valorise stoicism, self‐reliance and the minimisation of vulnerability (see, e.g., Ericsson et al. 2022; Farrimond 2011; Piatkowski et al. 2023; Seidler et al. 2016). Situated within this broader field of research, this section will thus elucidate the historical continuity through which intersecting constructions of masculinity may manifest and shape men's engagement with medical services.

As an introduction, we begin by presenting the story of Thomas, who was 61 years old, divorced, with three grown‐up children. He often worked shifts as a crane operator. When he was off shift, he mostly spent his time at home. Thomas talked about endurance as something close to a guiding principle in life—almost a moral compass. This emphasis on endurance, even in mundane situations, such as wearing ill‐fitting shoes for months without a single complaint, reflects not only a personal trait but also a culturally shaped idea of what it means to be a man, especially in working‐class settings (see also Taylor Smith and Dumas 2019).

It also points to how Thomas took up a masculine subject position that resists ageing: Despite being in his early sixties, he foregrounded resilience and bodily capability, reflecting a reluctance to identify with cultural notions of ageing or bodily decline (e.g., Thomas and Thurnell‐Read 2024; Twigg 2020). As Connell and Messerschmidt (2005) argue, (conventional) forms of masculinity are often organised around ideals such as toughness, self‐reliance and bodily control (see also Riska 2002). The same logic appears in Thomas's approach to his MI. He noticed increasing fatigue while cutting the grass and doing chores but chose to endure it for weeks before seeking help.

I went down to the health centre on Friday, and they were mainly going to take my blood pressure, because I thought that was what was wrong. I told the nurse what had happened, and she said, ‘Yes, but take it easy. We’ll check your blood pressure first, and then we’ll do an ECG’. She hooked me up and started looking at the readings, then she disappeared. ‘Yeah, but it’s okay’, she said when she came back. Then she returned with a doctor: ‘We’ll take you into another room’. So, I went in, and the doctor came in and said ‘Take it easy’. We’ve called an ambulance. You’re having a heart attack.

(Thomas, 61 years old)

Notably, Thomas’s reaction to the diagnosis lacks dramatisation. This emotional detachment may be read as a masculine connoted (and aged) performance, in which emotional control is upheld despite the life‐threatening situation. His account is illustrative of how our participants engaged in contacting healthcare. A large majority of those interviewed explained how they had formed an idea about what their condition was and that it would disappear by itself, thinking it was probably just a cold or the flu, or, as another participant, Eddie (68 years old), explained it, ‘I woke up and had this pain, and it was really bad. But I went and lay down on the couch because I figured it was heartburn. In my world, it was heartburn’. Even though Eddie was among the oldest participants in the study, his account suggests a refusal to align himself with cultural expectations of bodily decline and illness in older age.

These narratives reveal a tendency to minimise symptoms and reframe them in more mundane explanations, often delaying care in favour of maintaining daily routines and responsibilities. This also reflects broader gendered and classed logics: Seeking care prematurely could seem overly dramatic, contradicting an internalised ethos of stoicism and control (cf. Mahalik and Backus Dagirmanjian 2018). In this manner, severe symptoms (shortness of breath, severe chest pain etc.) are pitted against the intended path and routine of daily life. Another participant, Börje, who worked as a truck driver, explained:

Börje (50 years old) So, I was out driving the truck and talking to the fleet manager at the haulage company, and I felt totally fine, nothing at all. Then just a couple of minutes after the call, I suddenly felt like, ‘Damn, my chest really hurts’. And it was hard to lift my arm and stuff. And that's how it started.

Interviewer What did you do then?

Börje Well, I kept driving to where I was headed. It was about 60 km, maybe 65.

Interviewer But how did that go?

Börje Well… breathing was a bit tough, it was. But other than that, I didn't feel all that much.

Choosing to complete his delivery before seeking medical attention can, of course, be seen simply as a form of negligence and unawareness (see Ericsson et al. 2022). At the same time, this form of narration centres on working‐class values such as endurance, duty and dependability. For Börje, the body was a tool of labour, and stopping before completing a task felt (morally) unacceptable. What is exemplified here is thus a form of class‐based occupational masculinity (cf. Mahalik and Backus Dagirmanjian 2018), in which physical endurance and a striving not to disrupt the workday stand as markers of competence and integrity (at the expense of one's own health). This working‐class ideal of enduring pain to fulfil one's duty contrasts with what Springer and Mouzon (2011) describe as a more middle‐class form of masculinity, in which health neglect may be rooted less in occupational obligation and more in the performance of autonomy and invulnerability (see also Riska 2002).

Another of our informants, Olof, worked in business administration and development. He managed a portfolio of companies, providing strategic guidance, facilitating recruitment and coordinating external expertise as needed. As he had just turned 65, he talked about the future and his upcoming retirement. Having always been a runner, he had envisioned a new career as a senior long‐distance runner. He explained that he had always been very confident in his health due to a disciplined lifestyle and consistent exercise. The onset of his heart attack therefore came unexpectedly, to say the least. He felt shortness of breath and a recurring need to rest and some chest pain but dismissed these symptoms as not serious. His wife, however, was worried.

So, on that day, a Monday, I drove up to work and called my wife from there. She kept insisting I see a doctor. So I contacted my health insurance and got in touch with a doctor that way. I think he asked me like three questions, then just said: ‘Go to the ER. Now!’ (…) And I thought, ‘Well, I’ve got a bunch of meetings this morning, I’ll do those first and go later’. But then I changed my mind. He was a doctor, after all. So, I drove down there. And I had barely walked in and said, ‘Yeah, I’ve got some pain here’, when the woman at reception said: ‘Lie down. Don’t move’.

(Olof, 65 years old)

What surfaces here is a somewhat different, yet related, masculine subject position compared to the previous narrative given by Börje. Instead of occupational endurance, Olof exemplifies a self‐managed masculinity. At the same time, both men put off seeking care, albeit for somewhat different reasons: one due to duty, the other due to confidence in bodily control and adherence to professional commitments. In this sense, Olof's narrative can be understood as a distinctively middle‐class‐formed masculinity (i.e., self‐discipline, bodily control and forward planning, rather than endurance and manual labour). Indeed, his narrative centres around values such as autonomy, balance and the optimisation of health—hallmarks of a neoliberal health ideal in which responsibility for well‐being is individually managed (Henwood et al. 2011). Furthermore, the ‘disruption’ of having an MI is seemingly amplified by its timing, coinciding with his 65th birthday and the anticipated transition from professional life to a self‐defined late adulthood. His commitment to running, paired with a lifestyle framed around discipline and high‐functioning autonomy, contributed to a perceived invulnerability. The heart attack, therefore, did not only represent a biomedical event but also an existential disruption of a carefully cultivated masculine subject position and project.

What this section ultimately shows is how help‐seeking is delayed through gendered logics that cut across class and age. Despite their differences, the men articulated a shared temporal rhythm, one that preserves autonomy and control until the threshold for intervention becomes unavoidable. These thresholds are not only medical but also symbolic. Delaying care enabled the men to preserve valued masculine subjectivities that resisted both vulnerability and ageing. In doing so, they took up or resisted positions of young or old masculinities, negotiating bodily decline and the social meanings of ageing in ways that reflected their intersecting class and age locations. Thus, these patterns demonstrate how health behaviours, rather than being solely individual choices, are shaped by socially constructed and intersectional norms in the context of health, illness and subjectivity.

### 5.2 The Quest for a New Life Balance—(Re)constructing Intersectional Masculinities

Although everyday habits are widely recognised in research as significant risk factors for MI (Vrints et al. 2024), the participants made few explicit connections between a stressful lifestyle and their MI. Instead, hereditary factors were often easier to discuss as causes. However, after MI, some participants began to reflect more critically on their ways of life and the need to promote healthier living post‐MI. This process largely took the form of compromises rather than radical changes. Among the middle‐class men, for example, many had successful careers characterised by long working hours and sacrifices in family life, often acknowledging that the MI functioned as a reminder of their mortality.

Mats, 38 years old, married with two young children, provides a vivid example. Like Olof, who was introduced in the previous section, he is into running and likes to compete in different long‐distance races. Last year, however, things changed, when he ran a half marathon and ‘had a sudden cardiac arrest and a heart attack some 100 m before the finishing line’. He was acutely treated, and when he gained consciousness, he demanded to be allowed to finish the race. He was, of course, not allowed to do this and was immediately sent to the hospital. When it was time to be discharged, he received some advice from his doctor.

I was advised to tell our kids, especially our older daughter, what had happened. And she’s old enough to understand. She’s eleven, you know. We haven’t talked about the details, like how close it actually was. (…) Then I was home for a few days, but I went back to work pretty quickly. They recommended that, too. To return to as normal a life as soon as possible. Just staying at home, that doesn’t work for me. I’m not that kind of person.

(Mats, 38 years old)

In the excerpt, Mats initially projected a subject position tied to fatherhood, protection and a form of emotionally regulated care. He narrated an emotionally charged situation (explaining the illness to his child) but did so through a lens of reason, shielding his daughter from existential details. This reflects a caring positioning that balances emotional presence with restraint, shaped by ideals of being a rational and responsible father (Johansson and Andreasson 2017). This form of emotional engagement, after MI, is also expressed in his reflections on returning to physical training. Mats admitted that for a time, he was ‘terrified of doing anything that would raise my heart rate. Because then I'd think, “That's it, I'll die again”.’

Again, emotional awareness and thoughts about life and death became paramount. Articulated this way, in different capacities, was an embodied fear that momentarily disrupted Mats’s ‘ordinary’ high‐held ideals of an active, disciplined male body (cf. Liu et al. 2016). In a way, the image of the rational and controlled self gives way to a more present and vulnerable subject, in which bodily fragility and emotional presence become central. Yet, this emotional availability appears temporally situated, emerging in the immediate aftermath of the cardiac event, and perhaps also spatially linked to the hospital context and the proximity to care and professional advice. Upon returning home, Mats explained, he soon ‘went back to work’, because, as he explained, just staying at home ‘doesn't work for me’. Seemingly, the desire to resume what he considered to be normal routines became primary, signalling the return to a more habitual, performance‐oriented mode of being (cf. Elliot 2016). Put differently, the new attentiveness that was described initially soon folded into a broader script of ‘getting back to normal’. For Mats, the idea of ‘normal’ involved long work hours managing his furniture‐trading business, combined with responsibilities on a horse farm. The temporal unfolding of Mats's story thus illustrates how emotionally reflexive positions tied to fear, care and vulnerability can be fleeting and gradually replaced by or return to a more conventional masculine subject position or what Riska (2002) describes as the ‘hardy’ man. This is a middle‐class‐based masculinity, the core features of which are described as being ambitious, competitive and successful, all while—although in the case of Mats this is perhaps debatable—retaining an overall health awareness (see also Jbilou et al. 2019; Mokhwelepa and Sumbane 2025).

A lifestyle marked by long hours, high demands and limited rest was common among the middle‐class men interviewed. Many described navigating competing expectations—balancing professional responsibilities with family life—and negotiating health‐promoting intentions alongside behaviours potentially detrimental to their well‐being. This was exemplified in Carl, a former property manager and reserve officer in the Swedish military. Married and with two adult children, Carl had historically structured his life around discipline and physical activity. However, with age, he found that maintaining his former routines had become increasingly difficult. He recalled the onset of his MI during what had been an ordinary walk as ‘sweating and starting to feel cold and clammy’. When he visited the medical centre (the next day), he was informed that he was experiencing an ongoing heart attack. Reflecting on its possible causes, Carl offered an ambivalent account:

After the heart attack, I was accused of being a bit lazy, which I don’t think I’ve ever been in my life. I’ve trained hard. I’m a reserve officer in the coast guard, and I’ve done the famous Vasa cross‐country skiing race. But at 50, I thought I could stop training. I didn’t need that anxiety anymore. But with reduced activity and probably eating too much fatty food, my arteries clogged.

(Carl, 69 years old)

Carl acknowledged ongoing risky behaviours, including consuming four or five bottles of wine weekly. He was aware of the health risks and partially recognised the role of stress in his illness. He continues:

No, these problems probably wouldn’t have happened if I hadn’t pushed myself so much, if I’d understood that I shouldn’t overdo things, and if I hadn’t set such high goals for everything. I had to prove to the outside world, and myself, that I was good enough.

When reflecting on the factors contributing to his MI, Carl conveyed a sense of ambivalence about his health behaviours and lifestyle. He acknowledged a lifetime of physical activity and discipline but admitted that he had gradually let go of these routines. The shift was not necessarily towards better self‐care, but rather a relaxation of the bodily discipline that had previously defined his sense of self. Although he did not explicitly mention his children in the excerpt, his narrative reflects a form of breadwinner masculinity, anchored in performance, control and achievement, qualities that structured both his career and personal identity. With age, however, this position becomes harder to maintain. Yet, the underlying drive to prove oneself remains. In this way, Carl's story illustrates how ageing and masculinity intersect: Relaxing one's physical routines does not dissolve the desired masculine position but may instead give rise to contradictions between the desire for control, the ageing body's limitations and persistent behaviours that continue to jeopardise health.

Mats and Carl (discussed above) embody a hyperindividualistic approach to lifestyle and a work ethic that rarely mentions the family; rather, work and personal pleasures are emphasised. In the narratives of the participating working‐class men, however, a somewhat different picture appears. Johannes, 50 years old, a carpenter, married with two children, described his MI as sudden and unexpected. He underwent surgery and returned home, viewing the event as an ‘alarm clock’.

I’ve changed my diet, started to exercise, and reduced stress. What else can you do? You can’t stop living. You must try to make the best of it. And that’s what I’ve decided to do as long as I live. (…) My wife is a little more worried about me though. We haven’t talked much about it, but I notice she’s more cautious about things. Like she had a 30‐year‐old before, but now she’s got a 60‐year‐old.

(Johannes, 50 years old)

Johannes became more aware of his bodily fragility post‐MI. He experienced impotence and began to contemplate ageing and bodily changes differently, slowly adapting to his new physical condition. This expression of embodied vulnerability was narrated with humour and relationality, contrasting with the individualistic strivings of Carl and Mats. Johannes positioned himself within a masculinity that accepts slowing down, and the presence of his wife played a significant role in shaping his sense of self post‐MI. This is also mirrored in the narratives of others.

Johan, 75 years old, was a former priest, married with four children. After his MI, he had to rethink his role in the family.

I was the one handling everything practical, basically, especially after I retired and was living at home more. Then it really became my thing to manage [the family]. But they’ve handled everything while I’ve been unwell. So, I can’t just barge in and say, ‘Now I’m healthy and I’m taking charge again’. Instead, I’ve stepped back and told them, ‘You’ll have to take care of things now’.

(Johan, 75 years old)

In Johan's narrative we see how a masculine subject position can be gradually shaped by age, relational responsibility and a growing acceptance of dependency after illness. His willingness to step back from a formerly central and somewhat patriarchal role in the family reflects an adjustment not only to physical limitations but also to shifting generational dynamics and emotional openness. The presence and agency of his wife and adult children were central in this process, signalling a reconfiguration of authority and care within the family unit. When read alongside Johannes's narrative, a subtle classed dynamic begins to take shape. Although Johannes, a working‐class carpenter, centred his experience around physical vulnerability and the significance of his wife's presence post‐MI, Johan represented what perhaps could be described as a lower‐middle‐class position in which emotional availability and family adjustment are similarly foregrounded. In contrast to Mats and Carl—both situated in higher‐middle‐class, high‐performing roles—their stories contain more room for relationality, humour and embodied adaptation. Although these distinctions, of course, cannot be generalised across class categories, they illustrate how subject positions can be negotiated differently depending on one's socioeconomic position, age and life trajectory. In particular, they point to a discursive tension within middle‐class masculinities, in which hegemonic ideals of autonomy and control remain salient but may be more readily tempered by relational concerns and the embodied realities of ageing and illness. Taken together, these findings thus serve to extend our theoretical understandings of masculinities by showing how hegemonic and relational ideals are dynamically negotiated across age, class and family contexts, highlighting the intersectional shaping of masculinities after MI.

## 6 Conclusions and Discussion

Starting with constructs of masculinity, the analysis revealed how gendered understandings of illness and recovery moved between ideas of masculinity as a persisting structuring force and a dynamic and situated practice shaped both by temporal aspects of the illness and social location. Across the data, various configurations of masculinity were expressed, stretching from stoic endurance and bodily control to emotional reflexivity and relational responsibility. These configurations were not equally valued, however, by the study participants. Certain masculine ideals, such as autonomy, physical resilience and productivity, tended to remain dominant, whereas other modes, such as vulnerability or care orientation (for oneself and others), were more conditionally addressed by the participants. Yet, the stratification of these ideals was not static. They were negotiated, reasserted or reoriented, depending on where our participants found themselves in the trajectory of their illness. For example, although stoicism led the men in our study to postpone their care‐seeking during early symptom onset, showing a historical continuity in men's ways of seeking medical care, proximity to death during a cardiac event often disrupted this posture, creating temporary openings for other subject positions to surface. For some participants, these shifts were soon folded back into dominant logics of normalisation (like Mats); for others, this was less so, as when Johan engaged in a process of reconfiguring his understanding of masculinity, as well as his position in the family, by rejecting his former control over family matters and bringing forward ideals such as interdependence and relationality (see also Elliot 2016). Taken together, these findings extend our understanding of hegemonic masculinity by showing how dominant masculine ideals are both upheld and contested in the context of illness and recovery, highlighting the ways men negotiate, temporarily disrupt or reconfigure these ideals in response to changing bodily and experiential circumstances.

Importantly, mirroring Wemrell et al. (2017), the analysis also demonstrates how masculinity is deeply intertwined with classed and aged positions, foregrounding the relevance of intersectional perspectives in the sociology of illness. For example, our findings revealed that participants from a working‐class background often mobilised occupational masculinities rooted in endurance, duty and physical labour, whereas, by contrast, those from middle‐class backgrounds tended to perform a more individualised and self‐optimising masculinity, though often equally marked by delayed care‐seeking and a reluctance to appear vulnerable (cf. Jbilou et al. 2019).^1^ Age further served to shape these configurations. Among older participants, masculinity was increasingly negotiated in relation to bodily limitations, shifting family dynamics and generational roles. For some, this gave rise to a more relational and emotionally available subject position, in which authority was shared or relinquished in favour of care and interdependence (see also Willis et al. 2023). For others, ageing was resisted through attempts to sustain youthful, high‐functioning bodies and lifestyles, sometimes in contradiction of medical advice. Importantly, even though older men are often treated as a homogeneous and unmasculine category (Thomas and Thurnell‐Read 2024), this study illustrates that masculinity and age are continuously reconstructed and renegotiated throughout the life course. Similarly, it can be noted how classed positions serve to shape how masculinity is performed and experienced in the context of health and illness.

Although previous research has documented the gendered nature of MI experiences and help‐seeking behaviours (e.g., Emslie and Hunt 2009; P. M. Galdas et al. 2010; Smith et al. 2022), our study contributes by showing how such gendered health practices are stratified and negotiated through class and age, among other things (see also Wemrell et al. 2017). Moreover, it illustrates how masculine norms and ideals are not only externally imposed but also internalised and used as discursive resources to frame one's illness through narratives of discipline, sacrifice or emotional control. The dynamic nature of these subject positions also suggests that MI, while often reinforcing conventional notions of masculinity (as above), may at times function as a moment of rupture at which alternative ways of being a man are envisioned, even if temporarily. In doing so, the study offers a nuanced contribution to our understanding of the ongoing reconfiguration of hegemonic masculinity in contemporary Swedish society. This also has an impact on the understanding and planning of health promotion, as well as primary and secondary prevention of cardiovascular disease.

These insights carry implications for both research and healthcare practice. From a scholarly perspective, there is a continued need to unpack the relational and contextually embedded nature of masculinities in illness trajectories, not least considering shifting cultural ideals around health, gender, ageing and class. For healthcare professionals, the findings point to the importance of recognising how intersecting social positions influence how men (and women) interpret symptoms, engage with healthcare and respond to recommendations. Rather than pathologising a gendered tendency to postpone the seeking of medical help as irrational or culturally deficient, interventions might more effectively support men by acknowledging and problematising the moral and social logics that underpin this tendency. For example, health promotion campaigns and public health policies could frame rapid help‐seeking when experiencing symptoms of an acute MI as a practice that aligns with valued masculine ideals, such as responsibility, relational care for others or bodily competence. Such reframing may help challenge dominant norms that position illness disclosure as a sign of vulnerability. Given that patient delay can lead to poorer prognosis and increased complications after an MI, it is also important to raise awareness among both the public and healthcare professionals that older men and men in structurally disadvantaged socioeconomic positions may be particularly vulnerable to delaying care. These delays can be understood not only as individual choices but also as patterned responses shaped by gendered norms, classed expectations of stoicism and unequal access to resources. Recognising these gendered and sociostructural dynamics is therefore crucial for developing interventions that are both equitable and effective. Furthermore, relational dynamics, such as those with partners or adult children, appear relevant to many men's recovery, suggesting that more inclusive and family‐oriented models of care may support longer‐term health engagement. Finally, the study calls for increased attention to how classed and aged experiences of masculinity shape not only access to care but also the very meanings attached to health, vulnerability and recovery.

## Author Contributions

**Jesper Andreasson:** funding acquisition, conceptualization, investigation, methodology, formal analysis, writing – original draft, writing – review and editing. **Thomas Johansson:** conceptualization, formal analysis, writing – original draft, writing – review and editing. **Carina Danemalm‐Jägervall:** resources, data curation. **Anna Strömberg:** resources, data curation, writing – review and editing.

## Funding

This work was supported by Forte – Forskningsrådet för hälsa, arbetsliv och välfärd (2023‐00286).

## Ethics Statement

Formal ethical approval to carry out the study was given by the Swedish Ethical Review Authority (Ref. No. 2023‐07225‐01).

## Consent

The study was conducted in accordance with relevant ethical guidelines and was approved by the Swedish Ethical Review Authority (Ref. No. 2023‐07225‐01). All participants were fully informed, both verbally and in writing, about the purpose and design of the study, as well as their rights, including the right to withdraw at any time without consequences. Written informed consent was obtained from all participants prior to the interviews.

## Conflicts of Interest

The authors declare no conflicts of interest.

## Data Availability

Data sharing is not applicable to this article as no datasets were generated or analysed during this study.

## Endnotes

^1^
Even though this study focuses exclusively on men and their gendered subject positions in relation to help‐seeking and illness narratives, we acknowledge that delayed care‐seeking is not specific to men. Indeed, existing research has shown that women—particularly older women—also exhibit marked delays in seeking and accepting care for MI, often due to atypical symptom presentation, sociocultural expectations and differences in how their symptoms are recognised and acted upon compared with men (Babiolakis et al. 2022). Such findings, we argue, further underscore the structural and gendered nature of health inequalities and illustrate the relevance of including intersectional perspectives in both clinical practice and health research.
